# Deep brain stimulation for chronic pain: mechanisms, clinical applications, limitations, and future directions

**DOI:** 10.3389/fmed.2025.1683991

**Published:** 2025-10-27

**Authors:** Lou’I Al-Husinat, Majdala Al-Bataineh, Rama Hayajneh, Dania O. Haneyah, Qabas Alrawabdeh, Rasha Bani Melhem, Aiman Al Sharei, Mohammed I. Ismail, Saif Azzam, Sarah Al Sharie, Giustino Varrassi

**Affiliations:** ^1^Faculty of Medicine, Yarmouk University, Irbid, Jordan; ^2^Faculty of Medicine, Jordan University of Science and Technology, Irbid, Jordan; ^3^Azraq Refugee Camp Hospital, International Medical Corps, Amman, Jordan; ^4^Jordan Ministry of Health Hospitals, Amman, Jordan; ^5^Department of Pharmacology, Community Medicine, and Clinical Skills, Faculty of Medicine, The Hashemite University, Azzarqa, Jordan; ^6^Department of Anesthesiology, Mut’ah School of Medicine, Al-Karak, Jordan; ^7^Vanderbilt University Medical Center, Nashville, TN, United States; ^8^Fondazione Paolo Procacci, Rome, Italy

**Keywords:** deep brain stimulation, chronic pain, neuromodulation, neuropathic pain, periaqueductal grey

## Abstract

Chronic pain is a complex, multifactorial condition affecting millions worldwide, which is often resistant to conventional treatments. Deep brain stimulation (DBS), a reversible and adjustable neurosurgical intervention, has emerged as a promising therapeutic approach for the treatment of resistant chronic pain. This narrative review examines the evolving clinical role of DBS in pain management, highlighting its mechanisms, efficacy, limitations, and future directions. We discuss the neurophysiological underpinnings of chronic pain, emphasizing structural and functional changes in brain regions such as the medial prefrontal cortex, limbic system, and somatosensory pathways. DBS targets, including the periaqueductal/periventricular gray (PAG/PVG), the sensory thalamus, the anterior cingulate cortex (ACC), and the ventral striatum, are investigated in terms of pain modulation and affective processing. Clinical studies demonstrate significant variability in response rates, largely influenced by patient selection, lead placement, and pain etiology. While DBS shows the greatest success in nociceptive and neuropathic pain syndromes such as cluster headaches, phantom limb pain, and failed back surgery syndrome, its efficacy in deafferentation pain remains limited. Recent innovations, including dual-target stimulation, advanced imaging for surgical planning, and combination therapies with spinal cord or vagal nerve stimulation, offer promising avenues for improving outcomes. Despite its off-label status for pain in many countries, accumulating data support DBS as a viable treatment in select cases of intractable pain. Continued research and standardized protocols are essential for optimizing patient selection, refining targeting strategies, and improving long-term outcomes.

## Introduction

Chronic pain is a major and widespread public health concern worldwide, with a prevalence ranging from 8 to 50% (1). In the United States alone, it affects approximately 50 million adults and imposes an annual economic burden of US$560–635 billion, as reported by the Centers for Disease Control and Prevention (2). Individuals suffering from chronic pain may face difficulties in their personal relationships, experience reduced work efficiency, and deal with increased medical expenses (3). One of the earliest applications of DBS in functional neurosurgery was pain relief (1). DBS is a procedure that transmits electrical impulses to the brain via surgically implanted electrodes, which are placed in targeted brain regions using stereotactic methods and connected to an implantable pulse generator (IPG) (4). Common targets for chronic pain treatment include the sensory thalamus, periaqueductal gray (PAG) or periventricular gray matter (PVG), and the anterior cingulum (5). The appeal of DBS lies in its minimally invasive nature compared to other neurosurgical methods and its relatively high tolerability (6). According to the International Neuromodulation Society, neuromodulation is defined as the alteration of nerve activity through targeted delivery of a stimulus, such as electrical stimulation or chemical agents, to specific neurological sites in the body (5). Unlike earlier ablative methods that create irreversible brain lesions, DBS, as a form of neuromodulation, is both reversible and modifiable (6). DBS is widely used in movement disorders such as Parkinson’s disease (PD) and essential tremor (7). It has also been proven effective in epilepsy, cluster headache, Tourette’s syndrome, and obsessive-compulsive disorder (8). The concept of using DBS for intractable pain emerged in the 1950s, a decade prior to the development of the gate control theory (9). It is widely recognized that DBS can influence activity in both the lateral and medial pain systems (10). Reported success rates and clinical indications for DBS in pain syndromes are diverse and include conditions such as facial neuropathic pain, failed back surgery syndrome (FBSS), amputation pain, and brachial plexus injury (11). Additionally, DBS has been used effectively to treat pain caused by multiple sclerosis and spinal injuries (12). Variability in long-term outcomes can be attributed to factors such as differences in patient selection and medical conditions, inconsistent use of trial implants, variations in anatomical targets, and the duration of follow-up (13). While the primary focus of DBS research for chronic pain is often on pain intensity, the mood and neuropsychological components are also critical (14). Pain is multidimensional and includes sensory, cognitive, and affective aspects, with affective domains including pain-linked anxiety and fear (15). Certain DBS trials have targeted the neurocircuitry responsible for regulating affective pain, which can contribute to better emotional health for individuals with chronic pain. However, DBS was found to be more successful in alleviating nociceptive pain compared to deafferentation pain (16). The number of patients receiving DBS has increased by over 12,000 annually (17), being used off-label in multiple countries since the retraction of the Food and Drug Administration (FDA) approval after the late 1970s (18). As a result, despite its FDA approval for Parkinson’s disease in 2002, DBS for pain treatment remains off-label and is provided by only a limited number of neurosurgeons (19). In Europe, the use of DBS for chronic pain has been endorsed by the European Federation of Neurological Societies and approved by the United Kingdom National Institute for Health and Clinical Excellence (5).

A growing interest in DBS for chronic pain has been noted with an increasing amount of research over time. This narrative review provides a comprehensive overview and insight into the latest emerging trends regarding the clinical use of DBS.

## Chronic pain: mechanisms and challenges

### Pathophysiology of chronic pain

Chronic pain is a condition that persists beyond the normal healing process, becoming integrated with sensory and emotional brain regions such as the limbic system and the prefrontal cortex (20, 21). Neuroimaging studies demonstrate structural and functional changes in corticolimbic areas, shifting pain processing from sensory to emotional pathways, which contributes to pain chronification (20, 21). In addition, neurotransmitters and peripheral sensitization mechanisms, including glutamatergic activity and inflammatory mediators, further promote the persistence of pain (21, 22).

**Figure 1:**
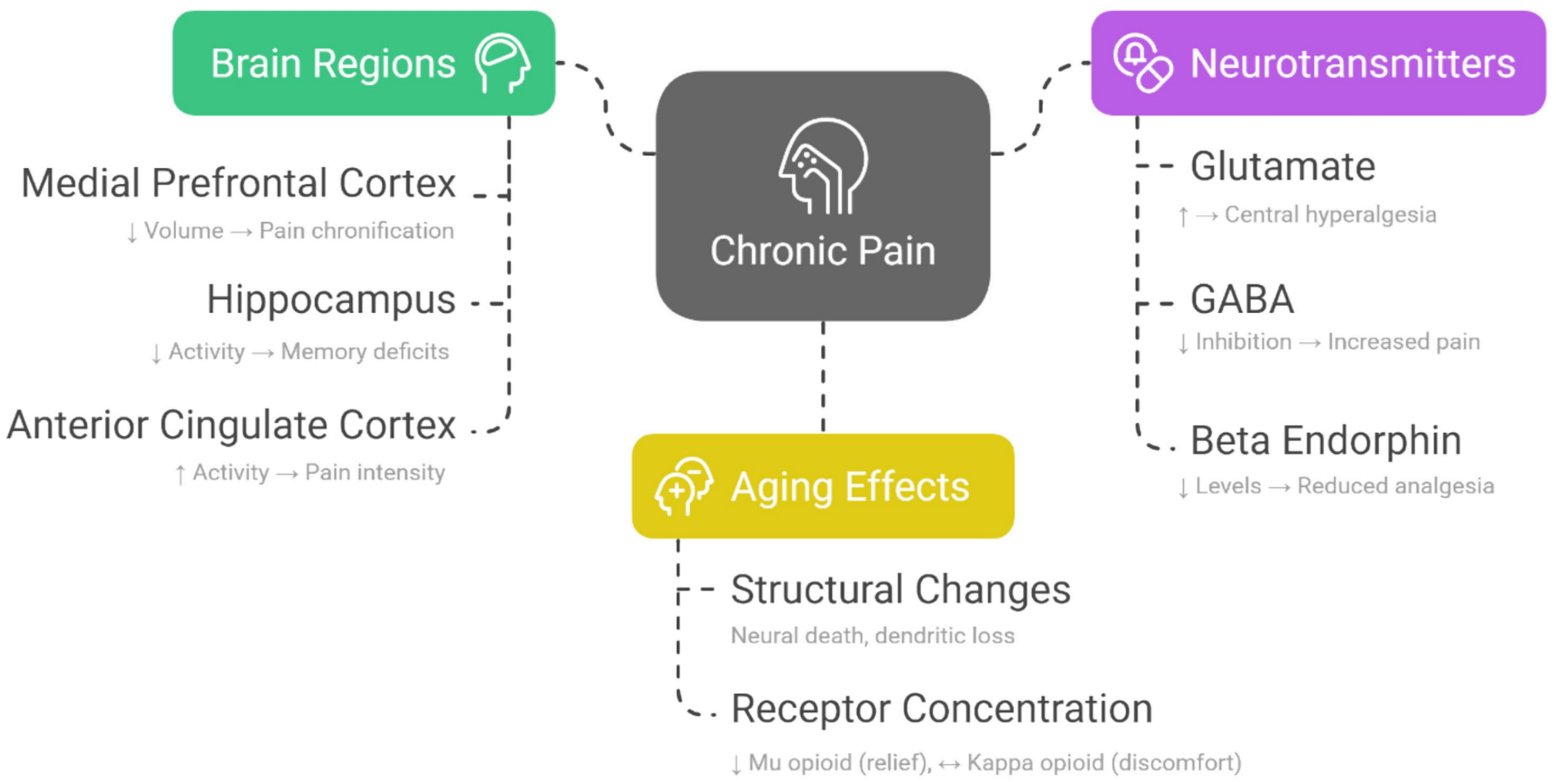
Chronic pain and its links to brain regions, neurotransmitters, and aging. Key brain areas show altered volume or activity, neurotransmitter imbalances heighten pain sensitivity, and aging exacerbates these effects through neural loss and reduced opioid receptor function.

Figure 1 provides a visual summary of the key brain regions, neurotransmitters, and aging-related factors involved in the pathophysiology of chronic pain.

### Types of chronic pain

In normal physiological conditions, after damage, there is a healing process, but in pathological cases, the pain can progress to the chronic stage, where it persists. Chronic pain is often idiopathic, though genetic susceptibility may play a role, particularly in cases following peripheral nerve injury (23).

Scientists have explained why some people develop chronic pain while others do not using the PSCEBSM model, where each letter represents a contributing factor: Pain, Somatic factors, Cognitive factors, Emotional factors, Behavioral factors, Social factors, and Motivation. The model helps in evaluating the factors that contribute to continuous pain (24).

Based on the International Association for the Study of Pain classification, chronic pain can be classified as nociceptive, neuropathic, and nociplastic pain (24). Neuropathic pain is characterized as a disorder of the nervous system (24). Chronic neuropathic pain can be divided into two categories: peripheral pain and central neuropathic pain. As the pain occurs as a result of an injury to the somatosensory nervous system, the patient must have had a previous injury to the nervous system or a diagnosis indicating such an injury (25).

Peripheral neuropathic pain usually occurs due to trauma, such as inflammation, toxins, and infections, which lead to damage in the peripheral nerves and are correlated to specific kinds of fibers, such as unmyelinated C-fibers, which are responsible for transmitting slow and continuous pain signals, and A-fibers, which are responsible for rapid and specific pain signals (23).

The diseases that affect one or more branches of the trigeminal nerve lead to orofacial neuropathic pain and are known as trigeminal neuralgia, which can be triggered by non-harmful stimuli such as touching or talking, and can also be classified further depending on the cause as idiopathic, classical, and secondary trigeminal neuralgia. The classic variant is the reason for vascular compression, whereas the secondary variant causes multiple sclerosis and an angle tumor or cyst (25).

Diseases such as DM, autoimmune diseases, viral or bacterial infections, and toxins may cause painful polyneuropathy, which causes the feeling of numbness and loss of sensation, mainly caused by chemotherapy drugs (25).

Central neuropathic pain is due to injury in the central nervous system, mainly in the somatosensory system, which leads to a total or partial loss of the pain or heat sensation, as well as hypersensitivity in the affected regions. This pain may occur spontaneously or can be evoked. It may be difficult to accurately determine the cause of central neuropathic pain when the effect of the pain appears months or years after the damage (23).

In some cases, spinal cord injury caused by damage to the somatosensory pathway causes chronic central neuropathic pain, and this damage might be due to mechanical trauma with hyperalgesia or allodynia (25). In other cases, trauma in the brain leads to injury in the somatosensory pathways, which results in a defect in sensory signal processing in the brain (25). Moreover, nociplastic pain is not related to the specific disease or lesion but rather is a defect in the central nervous system, and this type of pain is caused by stimulation of the pain receptors as a result of a disease or lesion, such as burns or inflammation in the joints (24).

Economic and psychosocial aspects of deep brain stimulation for persistent pain. The use of deep brain stimulation (DBS) is heavily influenced by psychosocial and economic issues in addition to the cognitive and affective aspects of chronic pain. Caretakers frequently need to provide patients with continuing psychological support and help, which might affect therapy compliance and overall results. Furthermore, the high expense of the process and device upkeep raises questions about accessibility and cost-effectiveness, emphasizing how crucial it is to weigh these aspects when thinking about DBS as a therapeutic option (26, 27).

### Limitations of traditional pain management approaches

Traditional pain management approaches, such as pharmacological treatments, physical therapy, and interventional procedures, often face significant limitations. Opioids, while effective for acute pain, carry risks of tolerance, dependence, and addiction, alongside side effects such as sedation and constipation (28). Non-steroidal anti-inflammatory drugs (NSAIDs) may cause gastrointestinal or cardiovascular complications with long-term use (29). Physical therapy and psychological interventions, though beneficial, are not always sufficient for severe or refractory cases (30). Interventional techniques such as nerve blocks or ablative surgeries may provide temporary relief but can lead to complications such as nerve damage or recurrence of pain (31). These limitations highlight the need for alternative therapies such as DBS in treatment-resistant chronic pain.

### Mechanism of action of DBS

DBS has shown effectiveness in treating chronic pain, particularly through motor cortex stimulation (MCS). Studies targeting various brain regions demonstrate its efficacy in conditions such as phantom limb pain, stump pain, failed back surgery syndrome, anesthesia dolorosa, and burning hyperesthesia (32).

In addition, imaging techniques have been used to understand patients’ responses to DBS, such as positron emission tomography (PET) scans using 11C-diprenorphine, which work by measuring the number of opioid receptors before surgery, and functional magnetic resonance imaging (fMRI), which helps in predicting the most likely candidates to be responsive to DBS. Additionally, diffuse tensor imaging is used to increase the accuracy in predicting clinical outcomes (32).

DBS is considered an invasive brain stimulation technique to treat chronic pain by sending an electrical current to the brain through putting electrodes in a specific area using stereotactic techniques. These electrodes are then connected to electrical pulse generators inside the patient’s body. After high-frequency stimulation, the neural cells near the electrodes will become inactive, while fiber pathways remain active (5).

DBS devices operate by generating electrical pulses from the IPG to stimulate targeted brain regions. However, electromagnetic interference can disrupt the function of DBS systems through three main mechanisms: inductive coupling, conductive coupling, and radiated energy transfer. Devices have been developed to minimize electromagnetic interference in DBS systems, including device shielding and improved programming protocols. Clinicians routinely evaluate DBS function using external programmers that allow for the real-time adjustment of stimulation parameters and verification of system integrity. The majority of the adjustments are performed on an outpatient basis without surgery, whereas surgical revision is only required in cases of hardware malfunction or battery depletion (33, 34).

Inductive coupling occurs when nearby magnetic fields interact with the device. Conductive coupling involves electrical currents transmitted through conductive materials, such as the metal leads connected to the device. Finally, radiated energy transfer refers to electromagnetic waves emitted by nearby devices that can interfere with the DBS system (33).

Additionally, there is a technique that involves implanting electrical electrodes guided by MRI, often combined with dual-site stimulation. This approach requires electrodes long enough to reach both the PAG and the centromedian-parafascicular complex, enabling the stimulation of all targeted regions (35).

A study involving seven patients with chronic cluster headache began with preoperative MRI imaging. Five patients received unilateral electrode implantation, while the remaining two received bilateral implants. After 12 months, voxel-based statistical analysis was conducted to identify the most effective stimulation sites. The results showed no surgical complications, and six of seven patients responded to the therapy. On average, there was a 76% reduction in overall headache burden, a 58% decrease in headache frequency, and a 51% reduction in attack duration. The proposed mechanism of action is that DBS may disrupt abnormal activity in the ventral tegmental area, leading to pain relief (36).

Despite its potential, DBS is still not approved in the United States due to insufficient efficacy demonstrated in clinical trials (5).

### Clinical evidence for DBS in chronic pain

To date, it remains unclear why some patients respond to DBS while others do not. Several factors may contribute to this variability, including differences in lead placement, stimulation settings, and individual neuroanatomy. Other possible reasons include variations in clinical characteristics, comorbidities, underlying pathophysiology, or the exact location of the implanted leads (37).

Several patient-related factors may also influence DBS outcomes, including the type of pain, patient characteristics, and adherence to self-care practices. The following are some illustrative examples: Patient section: For instance, patients with neuropathic pain often exhibit a more favorable response to DBS compared to those with nociceptive pain. This difference may be attributed to the distinct pathophysiological mechanisms underlying these pain types. Self-care section: factors such as patient age, comorbidities, and adherence to self-care routines can significantly influence treatment outcomes. For example, older patients or those with multiple health conditions may experience less pronounced benefits from DBS, highlighting the importance of personalized treatment plans (38).

For example, a study by Cheema et al. (37) investigated the effects of DBS targeting the ventral tegmental area (VTA) in 43 patients, all of whom were followed for at least 1 year (mean follow-up duration: 5.6 years). The study demonstrated a statistically significant reduction in median attack frequency, from 140 to 56 attacks per month (Z = −4.95, *p* < 0.001), a decrease in attack severity from 10/10 to 8/10 (Z = −4.83, *p* < 0.001), and a reduction in attack duration from 110 to 60 min (Z = −3.48, *p* < 0.001).

A study by Owen et al. (39) assessed the outcomes of DBS targeting both the sensory thalamus and the PVG/PAG complex. The results showed that PVG stimulation alone provided the greatest pain relief in 17 patients (53%), while combined PVG and thalamic stimulation was most effective in 11 patients (34%). Thalamic stimulation alone was optimal in only 4 patients (13%). Overall, PVG stimulation demonstrated the highest efficacy, with a mean pain reduction of 59% (p < 0.001), and 66% of patients experienced a ≥ 50% improvement in pain levels.

A study by Dellapina et al. (40) found that DBS increased the heat pain threshold in Parkinson’s disease patients with pain (from 40.3 ± 4.2 °C to 41.6 ± 4.3 °C, *p* = 0.03) and reduced pain-induced cerebral activity in the somatosensory cortex (Brodmann area 40). In contrast, no such effects were observed in pain-free patients. These findings suggest that subthalamic nucleus DBS elevates pain thresholds and helps restore the function of the lateral discriminative pain pathway in Parkinson’s disease patients experiencing pain.

In Plow et al. (41) study, different criteria were used. Instead of using the typical 50% response on the visual analog scale (VAS) as the success criterion, the choice of outcomes was based on the intended therapeutic benefit. Because the affective experience of pain, rather than pain intensity itself, may be influenced by DBS of ventral striatum/anterior limb of internal capsule (VS/ALIC), the Pain Disability Index (PDI) was chosen instead. A success criterion with this measure was defined as a 40% reduction.

Recent literature suggests that subthalamic nucleus low-frequency stimulation (STN LFS) may help in alleviating non-motor symptoms of Parkinson’s disease, particularly chronic pain. In a study by Belasen et al. (42), the authors demonstrated that LFS alters thermal and mechanical sensory detection more significantly than high-frequency stimulation (HFS). This finding indicates that LFS offers a novel approach for modulating chronic pain in Parkinson’s disease patients undergoing STN DBS. The authors proposed that STN LFS could become a viable treatment option for patients with pain as their primary symptom.

A study by Hacker et al. (43) showed that early DBS decreases the necessity for the complexity of PD medications; it also provides long-term motor benefits over standard medical therapy. Further investigation is required, and the Food and Drug Administration has approved the conduct of a prospective, multicenter, pivotal clinical trial of DBS in early-stage PD.

In line with previous literature, the majority of the studies assessing pain in Parkinson’s disease after STN-DBS did not specify whether evaluations were performed in the ON or OFF levodopa state. Evidence further suggests that pain relief is often associated with improvements in motor symptoms such as rigidity and dystonia, although some studies also indicate a direct modulatory effect of DBS on sensory and pain processing. Improvements are most consistently reported for musculoskeletal and dystonic pain, while the effects on neuropathic or central pain remain less consistent.

Alagapan et al. (44) study investigated patients with treatment-resistant depression (TRD). Many patients with TRD who attempted experimental subcallosal cingulate DBS have responded to continuous stimulation with permanent symptom alleviation. However, the management of these patients is complex due to interacting factors, so patients showed variable recovery; some achieved clinical response earlier than others.

In a study about DBS for depression by Sheth et al. (45), the authors found that depressed people who received DBS had an increase in interest in pleasurable activities and closer emotional connections to loved ones. Also notable was an increase in concentration and improvement in performance at work, as well as decreased performance anxiety when making presentations to colleagues and clients. Neuropsychological assessments conducted after open-label optimization showed enhancements in semantic fluency and abstract visual reasoning, in addition to short-term and long-term recall of non-contextual verbal information. However, it remains unclear whether these cognitive gains stemmed directly from stimulation or if they were secondary to mood-related improvements.

In a study by Holewijn et al. (46), comparing general anesthesia and local anesthesia for DBS, no significant difference in outcomes was observed between asleep and awake subthalamic nucleus DBS for advanced Parkinson’s disease. However, the “asleep” procedure was perceived as less burdensome by patients and was, on average, 26 min shorter than the awake procedure.

Gubler et al. (47), findings indicate that the brains of epilepsy patients are significantly more vascularized than those of patients with Parkinson’s disease or obsessive-compulsive disorder. This increased vascularity makes surgical planning for DBS more challenging and limits the use of multiple electrode trajectories.

According to the type of pain, clinical evidence indicates that the response to DBS varies depending on the type of pain and the underlying condition. In Parkinson’s disease, the most consistent improvements are observed in musculoskeletal and dystonic pain, likely secondary to reduced rigidity and abnormal posturing (40). Studies assessing sensory thresholds also suggest a modulatory effect of DBS on pain perception independent of motor improvement (42). In contrast, neuropathic pain (12) shows more favorable outcomes than deafferentation pain, such as thalamic pain syndrome (48), which remains more resistant to stimulation. These findings emphasize the need to specify both the type of pain and the patient population when evaluating the clinical benefits of DBS. Table 1 presents a summary of clinical evidence on DBS for pain management.

**Table 1:** Summary of clinical evidence on DBS for pain management.

Study/Author	Patient group	DBS target	Pain type	Key outcomes	Notes
Dellapina et al. (40)	Parkinson’s disease with chronic pain	Subthalamic nucleus (STN)	Musculoskeletal/central pain	Increased heat pain threshold (40.3 °C → 41.6 °C); reduced pain-related cortical activity	Analgesic effect independent of motor improvement
Belasen et al. (42)	Parkinson’s disease undergoing STN-DBS	STN (low vs. high frequency)	Chronic pain/Sensory thresholds	Low-frequency stimulation improved thermal and mechanical thresholds more than high-frequency stimulation	Suggests central modulation of pain pathways
Owen et al. (39)	Patients with mixed neuropathic pain	PAG/PVG and sensory Thalamus	Neuropathic pain	Mean 59% pain reduction; 66% achieved ≥50% improvement	PAG stimulation provided the greatest benefit
Cheema et al. (37)	Chronic cluster headache	Ventral tegmental area (VTA)	Cluster headache/facial pain	Attack frequency reduced (140 → 56 per month); 67% responders	Demonstrated long-term efficacy in headache reduction
Plow et al. (41)	Thalamic pain syndrome	Ventral striatum/anterior limb of internal capsule (VS/ALIC)	Deafferentation pain	Improved disability index (affective component), not pain intensity	Highlights effect on affective dimensions of pain
Boccard et al. (9)	Phantom limb pain and failed back surgery syndrome	Sensory thalamus and PAG	Neuropathic pain	40–60% mean reduction in VAS pain scores	Variable outcomes depending on etiology

## Technical aspects of DBS

DBS systems consist of several key components: implanted electrodes, extension wires, and an IPG that delivers electrical impulses to targeted brain regions (4, 33). Electrodes are surgically placed using stereotactic techniques, often guided by advanced imaging such as MRI or diffusion tensor imaging to enhance precision in targeting structures such as the sensory thalamus or PAG (32, 35). Surgical procedures may be performed under either local or general anesthesia, with studies showing comparable outcomes between the two approaches (46). Technological advancements have significantly improved DBS efficacy, including the development of closed-loop systems that adapt stimulation in real time based on neural feedback and directional leads that provide more focused modulation of specific brain pathways (4, 6). Innovations such as dual-target stimulation (e.g., combined PAG and thalamic targets) and improved safety protocols for electrode placement have further expanded therapeutic possibilities (35). However, challenges such as electromagnetic interference with DBS devices remain, and this interference can occur through inductive coupling, conductive coupling, or radiated energy transfer (33). These technical refinements continue to optimize DBS for chronic pain management, though individualized programming and careful patient selection remain critical for success (5, 39).

## Patient selection and outcomes

Patient selection for DBS in chronic pain management follows strict eligibility criteria, typically focusing on individuals with refractory conditions, such as neuropathic pain, failed back surgery syndrome, or cluster headaches, who have failed conventional therapies (5, 11). Key predictors of treatment success include accurate lead placement, appropriate target selection (e.g., PVG/PAG or sensory thalamus), and the absence of significant psychiatric comorbidities (13, 39). Clinical outcomes are measured through standardized pain scales, with studies reporting mean pain reduction up to 59% in responders (39). Beyond pain scores, DBS has demonstrated positive impacts on functional outcomes and quality of life, including improved mobility and reduced medication dependence, though the results vary based on pain etiology and individual patient factors (14, 16). Long-term follow-up data suggest sustained benefits in select patient populations, particularly those with well-defined nociceptive components to their pain (9, 37).

## Safety, risks, and limitations

While generally well-tolerated, DBS carries potential risks including hardware-related complications (e.g., lead migration or infection in 5–10% of cases) and neurological side effects such as paresthesia or speech disturbances when targeting sensory or limbic structures (19, 33). The procedure’s invasive nature necessitates careful consideration of surgical risks, particularly in patients with highly vascularized brain anatomy (47). Ethical considerations include the management of patient expectations given the therapy’s off-label status for pain in many regions and the psychological impact of device implantation (18, 19). Additional limitations involve the variable response rates across pain syndromes, with neuropathic pain generally showing better outcomes than deafferentation pain (5). These factors underscore the importance of comprehensive preoperative counseling and multidisciplinary evaluation to balance potential benefits against risks (5, 13).

## Emerging trends

At first, procedures such as cordotomy, midline myelotomy, thalamotomy, and cingulotomy were performed to interfere with ascending pain pathways. Due to the significant risk of irreversible neurological damage, the inability to adjust pain control, and the possibility of neuropathic pain or anesthesia dolorosa, those ablative surgeries have largely been supplanted by high-frequency electrical stimulation (31, 49, 50). In patients with chronic pain, the PAG/PVG and sensory thalamic nuclei are frequent targets for intervention (1, 51), and more recently, structures such as the ACC and the VS/ALIC were targeted to diminish the emotional aspects of pain (1). Advancements in imaging, refined techniques, enhanced targeting, and improved electrode placement safety have facilitated the exploration of additional targets for central neurostimulation in chronic pain treatment (52). Furthermore, correct target selection and careful surgical planning for lead placement within the brain are crucial for effectively treating those painful conditions (53).

Bergeron et al. (49), in their narrative review, presented the insula as a new anatomical target for DBS in individuals suffering from chronic pain. The insula seems to play a crucial role in the central integration and processing of pain signals, with its high-frequency electrical stimulation may help alleviate the sensory and emotional burdens associated with chronic pain.

Moreover, DBS serves as a treatment for refractory pain conditions such as neuropathic pain, deafferentation pain, brachial plexus avulsion pain, chronic low back pain (CLBP), failed back surgery syndrome, and cluster headaches (52). Overall, conditions that tend to show a better clinical response include complex regional pain syndrome, phantom limb pain, and peripheral neuropathies (54, 55).

Data exhibited by Qassim et al. (56) concluded that facial pain was successfully reduced with a relatively low complication rate by the use of DBS, potentially influenced by placebo and lesion effects. MCS has been proven to be a viable therapy for chronic facial pain (5, 56, 57). However, DBS serves as an alternative, while more invasive, option for chronic facial pain treatment when MCS offers limited reactivity.

A newer emerging approach has explored the use of combination therapies as a treatment method. The combination of spinal cord stimulation (SCS) and DBS of the PVG has shown promising outcomes in treating FBSS. By adjusting stimulation parameters independently and concurrently, SCS effectively managed neuropathic leg pain, while DBS targeted nociceptive back pain (58). Furthermore, vagal nerve stimulation being utilized with DBS has been reported as a treatment for cluster headaches (59). Additionally, the combination of supraorbital stimulation with DBS has also been used for the management of cluster headaches (60).

## Conclusion

Deep brain stimulation offers a promising treatment for refractory chronic pain by targeting key brain regions such as the sensory thalamus and PAG/PVG. While studies show significant pain reduction in conditions such as neuropathic pain and cluster headaches, outcomes vary due to differences in patient selection, lead placement, and stimulation parameters. Emerging approaches, including new targets (e.g., insula) and combination therapies, may improve efficacy. Despite its invasive nature and off-label use, DBS provides adjustable pain relief where traditional treatments fail. Further research is needed to optimize protocols and expand its clinical role in chronic pain management.
